# Language uncovers visuospatial dysfunction in posterior cortical atrophy: a natural language processing approach

**DOI:** 10.3389/fnins.2024.1342909

**Published:** 2024-02-06

**Authors:** Neguine Rezaii, Daisy Hochberg, Megan Quimby, Bonnie Wong, Scott McGinnis, Bradford C. Dickerson, Deepti Putcha

**Affiliations:** ^1^Frontotemporal Disorders Unit, Department of Neurology, Massachusetts General Hospital and Harvard Medical School, Boston, MA, United States; ^2^Center for Brain Mind Medicine, Department of Neurology, Brigham and Women’s Hospital, Boston, MA, United States; ^3^Athinoula A. Martinos Center for Biomedical Imaging, Massachusetts General Hospital, Charlestown, MA, United States; ^4^Alzheimer’s Disease Research Center, Massachusetts General Hospital, Charlestown, MA, United States

**Keywords:** posterior cortical atrophy, language indicators, visuospatial impairment, semantic processing, natural language processing

## Abstract

**Introduction:**

Posterior Cortical Atrophy (PCA) is a syndrome characterized by a progressive decline in higher-order visuospatial processing, leading to symptoms such as space perception deficit, simultanagnosia, and object perception impairment. While PCA is primarily known for its impact on visuospatial abilities, recent studies have documented language abnormalities in PCA patients. This study aims to delineate the nature and origin of language impairments in PCA, hypothesizing that language deficits reflect the visuospatial processing impairments of the disease.

**Methods:**

We compared the language samples of 25 patients with PCA with age-matched cognitively normal (CN) individuals across two distinct tasks: a visually-dependent picture description and a visually-independent job description task. We extracted word frequency, word utterance latency, and spatial relational words for this comparison. We then conducted an in-depth analysis of the language used in the picture description task to identify specific linguistic indicators that reflect the visuospatial processing deficits of PCA.

**Results:**

Patients with PCA showed significant language deficits in the visually-dependent task, characterized by higher word frequency, prolonged utterance latency, and fewer spatial relational words, but not in the visually-independent task. An in-depth analysis of the picture description task further showed that PCA patients struggled to identify certain visual elements as well as the overall theme of the picture. A predictive model based on these language features distinguished PCA patients from CN individuals with high classification accuracy.

**Discussion:**

The findings indicate that language is a sensitive behavioral construct to detect visuospatial processing abnormalities of PCA. These insights offer theoretical and clinical avenues for understanding and managing PCA, underscoring language as a crucial marker for the visuospatial deficits of this atypical variant of Alzheimer’s disease.

## Introduction

Posterior cortical atrophy (PCA) is a clinico-radiological syndrome characterized by a progressive decline in higher-order visuospatial processing with relative preservation in other cognitive domains at initial presentation (Benson et al., 1988; Renner et al., 2004; Crutch et al., 2017). Common visuospatial symptoms of the syndrome include impaired object and space perception, simultanagnosia, environmental agnosia, and visual field defects (Crutch et al., 2017). From the neuroimaging perspective, the syndrome is associated with atrophy, hypometabolism, and usually tau deposition in posterior parietal, occipital, and temporo-occipital cortices (Whitwell et al., 2007; Lehmann et al., 2011). As the majority of PCA cases are due to underlying Alzheimer’s pathology, PCA is also referred to as the visual variant of Alzheimer’s disease (AD) (Levine et al., 1993; Alladi et al., 2007). While the diagnostic criteria for PCA indicate preserved functions in cognitive domains outside of visuospatial processing at symptom onset, a growing literature has documented language abnormalities in PCA emerging early in the course of the illness. Specifically, impaired category fluency and confrontation naming have been documented on formal neuropsychological assessments (Tang-Wai et al., 2004; McMonagle et al., 2006; Putcha et al., 2018, 2020). Language abnormalities are also evident during spontaneous speech, such as using higher frequency words and slowed speech rate (number of words per minute) (Crutch et al., 2013). These emerging observations suggest that there is still much to be understood about the nature and origin of language impairments in PCA.

The specific types of language abnormalities observed in PCA may be related to the network dysfunction that supports lexicosemantic retrieval, as has been previously postulated across the phenotypic spectrum of AD (Putcha et al., 2020). Another possible explanation is that the language abnormalities observed in PCA may stem from the visuospatial impairments central to the syndrome, rather than representing a primary language deficit. A large body of research supports close relationships between the visual processing of objects and the amodal semantic processing required for retrieving the names of those objects (Binder and Desai, 2011; Huth et al., 2016; Aliko et al., 2023). Recently, it has been shown that anterior to each region that is selective for the visual processing of a given category in the visual cortex, there is a corresponding area selective to its linguistic processing (Popham et al., 2021). This anatomical and functional configuration suggests that the anterior border of the visual cortex acts as a convergence zone where information from the unimodal visual system enters the amodal linguistic systems involved in linguistic retrieval. Therefore, the successful production of a word that has visual attributes requires intact visual processing, essential for providing the information needed to retrieve its corresponding linguistic representation (i.e., its name). Therefore, deficits in visual processing would theoretically impede the production of words with visual attributes. If the pathophysiology of language abnormalities in PCA involves disrupted visual processing, then it stands to reason that tasks heavily dependent on visual processing will exhibit significant language impairments. Conversely, tasks with minimal reliance on visual input should result in relatively intact language performance.

In the current study, we sought to test this hypothesis by contrasting the language used in two different speech samples as PCA participants described the Picnic scene from the Western Aphasia Battery (Kertesz et al., 2007) (visually dependent) and their prior jobs (visually independent). In each speech sample, we measured word frequency, word utterance latency, and the use of spatial relational words. For the picture description task, we hypothesized that PCA patients would use higher frequency words (e.g., replacing specific names of pictured items with superordinate words potentially including “thing”), have increased word utterance latency due to object recognition difficulty, and use fewer spatial relational words such as “into” or “underneath” compared to healthy individuals. For the non-visually dependent job description task, we expected these linguistic features to be comparable between PCA patients and healthy individuals.

Building on the hypothesis that speech patterns in visually dependent tasks reflect visuospatial processing deficits, we next sought to identify linguistic markers of these challenges. Specifically, we investigated which elements in the picnic scene presented particular retrieval difficulties and whether PCA patients could intuitively grasp and articulate the overall theme of the scene, such as using the word “picnic.” Due to the difficulty with visually integrating a scene (simultanagnosia) (Tang-Wai et al., 2004; Singh et al., 2015; Cui et al., 2022), we hypothesized that PCA patients were less likely to verbalize the term “picnic” compared to healthy individuals. Lastly, to address the clinical significance of this work, we used the language features derived from the picture description task to develop a classifier aimed at distinguishing PCA patients from healthy individuals and hypothesized a high degree of classification accuracy.

## Methods

### Participants

#### PCA patients

Twenty-five patients diagnosed with PCA were recruited from the Massachusetts General Hospital (MGH) Frontotemporal Disorders Unit PCA program for this study (Wong et al., 2019). All but one was confirmed amyloid positive (A+) and tau positive (T+) by either CSF analysis or amyloid and tau PET. The remaining participant’s biomarker status is unknown due to a failed lumbar puncture. Each patient had posterior cortical atrophy and/or hypometabolism (see Putcha et al., 2019 for the atrophy map of our PCA cohort), consistent with the typical neurodegeneration (N+) of PCA. All participants received a standard clinical evaluation comprising a structured history obtained from both participant and informant, comprehensive neurological and psychiatric history, as well as neuropsychological assessment. See Table 1 for neuropsychological profiles of the PCA cohort included in this study. Clinicians determined the impaired performance of PCA patients using the available normative data on these tests (Humphreys and Riddoch, 1993; Delis et al., 2000; Stern and White, 2003; Herrera-Guzmán et al., 2004; Strauss et al., 2006; Shirk et al., 2011; Weintraub et al., 2018). The clinical formulation was performed through a consensus conference by our multidisciplinary team of neurologists, psychiatrists, neuropsychologists, and speech and language pathologists, with each patient classified based on all available clinical information as having a 3-step diagnostic formulation of mild cognitive impairment or dementia (Cognitive Functional Status), a specific Cognitive-Behavioral Syndrome, and a likely etiologic neuropathologic diagnosis (Dickerson et al., 2017). Patients underwent neuroimaging sessions involving structural MRI, FTP PET, and amyloid (PiB or FBB) PET scans. Aβ positivity was determined by a combination of visual read and mean amyloid PET signal extracted from a cortical composite region of interest according to previously published procedures (Rabinovici et al., 2010; Villeneuve et al., 2015; Cho et al., 2023). Determination of tau positivity and neurodegeneration was conducted by visual read using internal methods similar to published work (Rabinovici et al., 2011; Fleisher et al., 2020; Sonni et al., 2020). This work was carried out according to The Code of Ethics of the World Medical Association (Declaration of Helsinki) for experiments involving humans. All participants and their caregivers provided informed consent in accordance with the protocol approved by the Mass General Brigham Human Research Committee Institutional Review Board in Boston, Massachusetts. A speech sample for the picture description task was acquired from all 25 PCA participants. Twenty-one PCA participants also took part in the job description task.

**Table 1:** Neuropsychological test data in Aß + posterior cortical atrophy (PCA).

Test	Mean ± SD
*Executive functions*	
Longest digit span forward	6.0 ± 1.6
Longest digit span backward	3.3 ± 1.0
Auditory addition (/12)	8.6 ± 3.5
Auditory subtraction (/12)	6.4 ± 3.8
Trail making test part A (seconds)	161.4 ± 59.0
Trail making test part B (seconds)	195.0 ± 53.7
*Language*	
Boston naming test (/30)	18.6 ± 7.6
Boston naming test with phonemic cue (/30)	24.1 ± 11.7
Auditory naming test (% correct)	88.6 ± 18.0
Auditory naming test with phonemic cue (% correct)	95.0 ± 10.2
Letter fluency (FAS)	35.0 ± 18.2
Category fluency (Animals)	12.3 ± 5.1
*Memory*	
Craft story immediate recall (/44)	10.3 ± 5.6
Craft story delayed recall (/44)	8.0 ± 5.6
CVLT-II-SF total recall (/36)	18.4 ± 7.0
CVLT-II-SF SDFR (/9)	4.2 ± 3.3
CVLT-II-SF LDFR (/9)	3.3 ± 3.1
CVLT-II-SF LDCR (/9)	3.6 ± 2.8
*Visuospatial*	
BORB single object identification (/40)	28.8 ± 7.7
BORB overlapping object identification (/40)	12.6 ± 8.2
VOSP number location test (/10)	3.8 ± 2.5
NAB visual discrimination (/18)	8.5 ± 3.4

As a group, our PCA cohort demonstrated significant impairment in visuospatial functions and memory encoding and retrieval. Milder impairment was observed in specific language and memory tests. Performance on tests of simple attention (longest digit span forward) as well as auditory naming with phonemic cues was intact. CVLT-II-SF, California Verbal Learning Test- 2nd Edition – Short Form. SDFR, short delay free recall; LDFR, long delay free recall; LDCR, long delay cued recall; BORB, Birmingham object recognition battery; VOSP, visual object spatial perception test.

#### Cognitively normal individuals

Twenty-nine cognitively normal (CN) participants (CN1) were enrolled through the Speech and Feeding Disorders Laboratory at the MGH Institute of Health Professions to participate in the picture description task. These participants passed a cognitive screen, were native English speakers, and had no history of neurologic injury or developmental speech/language disorders. Twenty-two CN participants (CN2) were additionally recruited through Amazon’s Mechanical Turk (MTurk) to describe their jobs. MTurk participants filled out the short and validated version of the 12-item Everyday Cognition questionnaire, a questionnaire designed to detect cognitive and functional decline (Tomaszewski Farias et al., 2011). Only language samples from participants who were native English speakers with no self-reported history of brain injury or speech/language disorder, either developmental or acquired, were included in the analyses. Table 2 compares demographic data across patients with PCA and CN groups. There was no statistical difference in any demographic data between CN1 and CN2. CN groups were matched to PCA patients with respect to age, gender, and handedness. We included healthy individuals if they had at least 12 years of education which resulted in PCA participants having higher average years of education than CN1 [*t*(33.47) = −3.07, *p* = 0.004] and CN2 [*t*(39.13) = −3.42, *p* = 0.002]. To ensure that the difference in years of education did not confound our results of language analyses, we conducted two additional analyses as presented in the Supplementary materials. We found no correlations between the language variables of interest and years of education. Furthermore, we observed findings similar to our main results when we repeated the analyses in a subgroup of PCA patients and CN participants with matched years of education (see Supplementary materials).

**Table 2:** Clinical characteristics of the Aß + posterior cortical atrophy (PCA) and cognitively normal (CN) group.

Demographics	PCA (N = 25)	CN1 (N = 29)	CN2 (N = 22)
Age (years)	68.4 ± 7.8	65.3 ± 8.4	66.9 ± 7.0
Sex (M/F)	13/12	13/16	7/15
Education (years)	17.2 ± 2.1	15.7 ± 1.0	15.2 ± 1.7
Handedness (R/L/Ambidextrous)	23/0/2	21/6/2	19/2/1
MoCA	14.6 ± 7.8		
CDR	CDR 0 (N = 1) CDR 0.5 (N = 11) CDR 1 (N = 10) CDR 2 (N = 3)		
CDR-SOB	4.5 ± 3.1		

Means and standard deviations are reported for continuous variables. MoCA, Montreal cognitive assessment; CDR, clinical dementia rating scale; SOB, sum of box scores.

### Speech samples and data analysis

Speech samples were collected under two conditions. For the visually dependent task, participants described the Western Aphasia Battery—Revised (WAB-R) (Kertesz et al., 2007) Picnic Scene with the instruction to use full sentences. For the visually independent task, participants were asked to describe what they did for work. There were no time limits applied to either task. Autotranscription was done using Google Cloud Speech-to-Text API for audio transcription (Cloud Speech-to-Text API v1 - Package cloud, n.d.), and manually verified by a research staff blinded to the diagnosis.

#### Speech sample analysis

All feature extraction was performed automatically using Quantitext, a fully automated speech and language analysis toolbox we developed in the Frontotemporal Disorders Unit of Massachusetts General Hospital. The program provides an objective assessment of language to enhance the precision of clinical evaluations as described previously (Rezaii et al., 2022). The program receives audio samples from participants and employs automated techniques for transcription, such as the Google API, ensuring reliable transcription accuracy. The toolbox uses a variety of software packages, such as Stanza (Qi et al., 2020) and Librosa (McFee et al., 2015) to conduct speech and language analysis. The program generates a comprehensive set of text-based metrics, including the ratio of parts of speech and dependency relations to the total word count, word frequency, syntax frequency (Rezaii et al., 2022, 2023a), content units (Josephy-Hernandez et al., 2023), total units, and efficiency of words (Rezaii et al., 2023c). Additionally, Quantitext assesses audio-based features encompassing the three primary domains of time, frequency, and time-frequency. The variables used in this work are further described below.

#### Word frequency

To measure word frequency, we used the Switchboard corpus (Godfrey et al., 1992), which consists of spontaneous telephone conversations averaging 6 min in length spoken by more than 500 speakers of both sexes from a variety of American English dialects. We use this corpus to estimate word frequency in spoken English, independently of the patient and control sample. The corpus contains 2,345,269 words. Here, word frequency denotes the log frequency of content words (comprised of nouns, verbs, adjectives, and adverbs).

#### Word utterance latency and articulation rate

Our analysis employed the Google Cloud Speech-to-Text API to ascertain word timestamps, pinpointing the onset and offset for each spoken word within the audio recordings. Speech rate—often quantified as the number of words spoken per minute—can vary based on factors such as word utterance latency and the individual articulation rate of each word. To ensure a more granular and accurate interpretation of the underlying phenomena, we sidestepped aggregated metrics like speech rate, focusing instead on separately evaluating its constituent components. Word utterance latency is defined as the time interval preceding the articulation of a word. This method was applied on all except for the very first word in each sample, as the time to start the description task depends on multiple factors. Articulation rate measures the number of syllables per second (Cordella et al., 2019).

#### Spatial relational words

Relational words are automatically tagged by Stanza as “case.” For most words, the relational words are spatial, for example, the word “under” in the sentence “I found the gem under my bed.” In our analysis, we divided the number of relational words by the total words.

#### Content units

To determine which items within the picture posed greater challenges for PCA compared to CN participants, we coded the visual items using content units. Content units are words with correct information units that are intelligible in context and accurate about the picture or topic. Words do not have to be used in a grammatically correct manner to be counted as content units (Nicholas and Brookshire, 1993). Each content unit is only counted once, regardless of how many times it is mentioned in a sample. The morphological variants were grouped within one single content unit. For example, the nouns “girl” and “daughter” are considered the same content unit. Therefore, if one participant used both words (girl and daughter), they would only be counted as one content unit. To specify content units, Quantitext first generates a Python dictionary using a predefined set of words as previously described and then uses this dictionary to automatically identify all content units in new texts it receives. Previously, we showed that the program has an accuracy of 99.7% in identifying content units (Josephy-Hernandez et al., 2023).

### Statistical analysis

We used Welch Two Sample t-tests to compare the language features across the two groups. We performed point-biserial correlation analysis on our dataset to investigate the relationships between the likelihood of reporting each content unit and the group designation (with PCA coded as 1 for patients and 0 for healthy controls). We applied Bonferroni’s correction to account for multiple comparisons, setting the significance threshold at 0.0016 (Bonferroni, 1935). For classification, we used a binary logistic regression model. We employed a leave-one-out cross-validation (LOOCV) approach on our dataset to validate the model’s performance. In each iteration of the LOOCV, a single observation was set aside as the test data, and the remaining observations were used to train the model.

## Results

### Language abnormalities in PCA are observed during picture description but not job description

**Figure 1:**
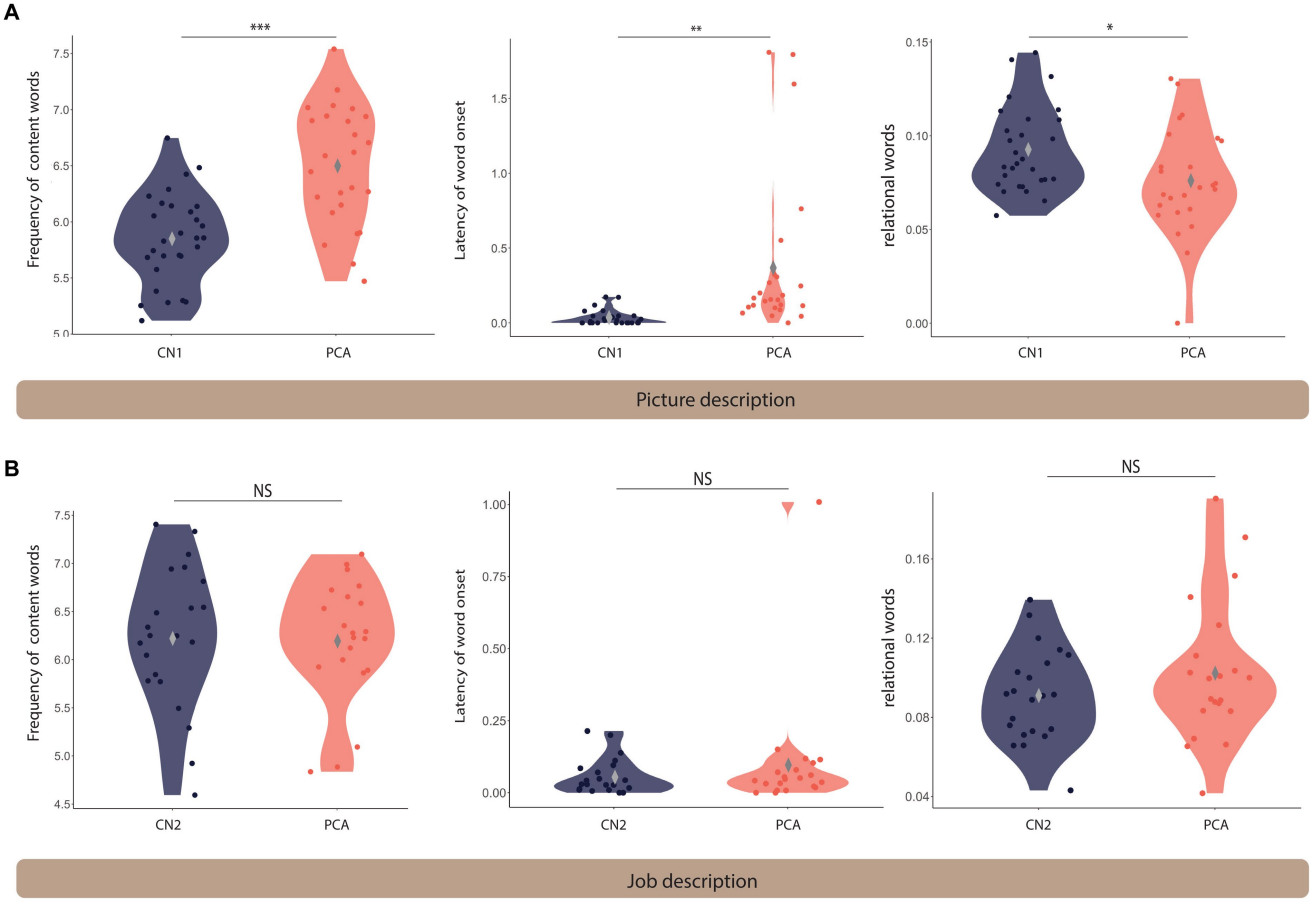
Language differs between PCA and healthy controls on the picture description task but not the job description task. Violin plots comparing the language features extracted from the picnic scene **(A)** and job **(B)** description tasks across healthy controls and PCA patients. *** denotes *p* < 0.001, ** indicates 0.001 < *p* < 0.01, and * shows 0.01 < *p* < 0.05. NS indicates not significantly different. The gray diamond indicates the m.

We first compared the speech samples of PCA participants describing the WAB Picnic Scene to the CN1 group to determine language abnormalities in this task. We also compared the speech sample from the job description task between the PCA and CN2 groups (see Table 3). We used Welch Two Sample t-tests to compare the means of the following features across the two groups (Figure 1).

**Table 3:** Comparing language features obtained from picture description and job description tasks across PCA patients and healthy individuals.

**Picture description**	**PCA**	**CN1**	***t*-statistics, *p*-value**
Word frequency	6.50 ± 0.53	5.85 ± 0.40	*t*(44.28) = −5.02, p < 0.001
Word onset latency	0.38 ± 0.54	0.03 ± 0.05	*t*(24.34) = −3.20, *p* = 0.004
Spatial relational words	0.08 ± 0.03	0.09 ± 0.02	*t*(45.55) = 2.35, *p* = 0.023
**Job description**	**PCA**	**CN2**	
Word frequency	6.20 ± 0.64	6.22 ± 0.74	*t*(40.66) = 0.13, *p* = 0.901
Word onset latency	0.10 ± 0.21	0.06 ± 0.06	*t*(23.42) = −0.83, *p* = 0.415
Spatial relational words	0.10 ± 0.04	0.09 ± 0.02	*t*(34.18) = −1.27, *p* = 0.213

#### Picture description task

Patients with PCA used higher frequency words (i.e., more commonly used words) (mean = 6.50 ± 0.53) compared to healthy controls (mean = 5.85 ± 0.40) [*t*(44.28) = −5.02, *p* < 0.001]. The time latency to the onset of words was longer for patients with PCA (mean = 0.38 ± 0.54) compared to healthy controls (mean = 0.03 ± 0.05) [*t*(24.34) = −3.20, *p* = 0.004]. We found that patients with PCA used fewer spatial relational terms (mean = 0.08 ± 0.03) compared to healthy controls (mean = 0.09 ± 0.02) [*t*(45.55) = 2.35, *p* = 0.023].

There was a trend toward a slower articulation rate in PCA patients (mean = 2.92 ± 0.60) compared to healthy controls (mean = 3.14 ± 0.35) [*t*(33.88) = 1.616, *p* = 0.115], suggesting that slower speech rate may be primarily due to an increased word utterance latency rather than articulation rate.

#### Job description task

There was a trend toward a slower articulation rate in PCA patients (mean = 6.20 ± 0.64) compared to healthy controls (mean = 6.22 ± 0.74) [*t*(40.66) = 0.13, *p* = 0.901]. Similarly, there was no significant difference in word utterance latency between PCA patients (mean = 0.10 ± 0.21) and healthy controls (mean = 0.06 ± 0.06) [*t*(23.42) = −0.83, *p* = 0.415]. No statistical difference was found in the use of relational words between PCA patients (mean = 0.10 ± 0.04) and healthy individuals (mean = 0.09 ± 0.02) [*t*(34.18) = −1.27, *p* = 0.213]. There was no difference in articulation rate in PCA patients (mean = 2.84 ± 0.45) compared to healthy controls (mean = 3.12 ± 0.45) [*t*(37.91) = 1.82, *p* = 0.077].

### Specific language indicators of visuospatial processing deficits of PCA can be extracted from the picture description task

**Figure 2:**
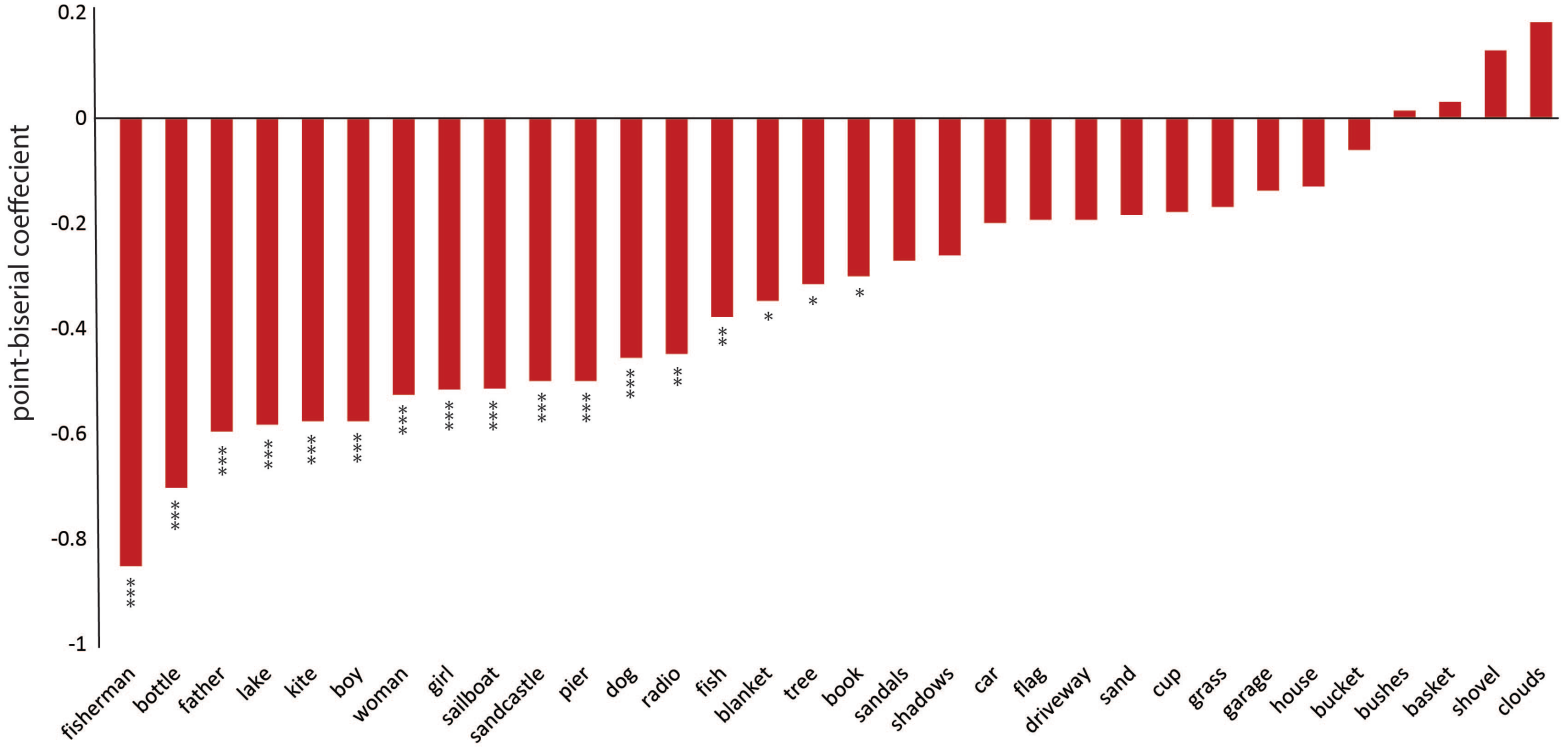
PCA participants and healthy individuals described different content units on the picture description task. The point-biserial correlation coefficients between the likelihood of reporting each content unit in the picnic scene and the designated group. Negative values indicate that PCA patients have a lower chance of mentioning the content unit compared to healthy individuals. *** denotes *p* < 0.001, ** indicates 0.001 < *p* < 0.01, and * shows 0.01 < *p* < 0.05. Bars without an asterisk are not significantly different.

**Figure 3:**
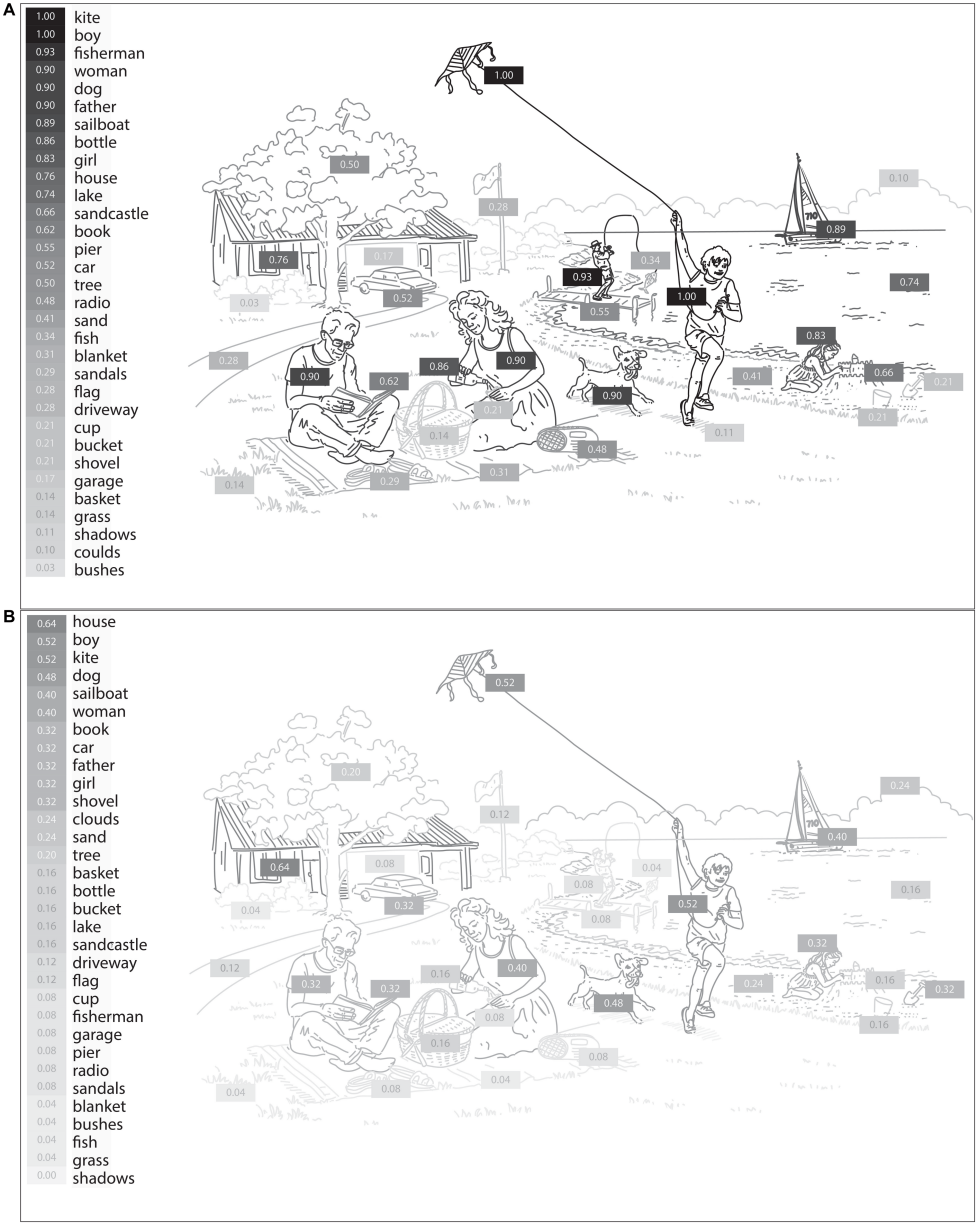
The likelihood of mentioning each content unit of the WAB Picnic Scene by healthy individuals **(A)** and PCA patients **(B)**. The shading intensity of each item corresponds to its verbalization probability by participants, with darker elements indicating a higher likelihood of being mentioned by PCA patients and healthy individuals.

We next probed the samples obtained from the picture description task to extract the specific language features that reflect visuospatial impairment in PCA compared to healthy controls. First, we determined the likelihood of mentioning each content unit by each diagnostic group. The picture consists of 32 content units, as shown in Figure 2. We performed point-biseral correlation analysis on our dataset to investigate the relationships between the likelihood of reporting each content unit and the group designation. As shown in Figure 2, we did not observe a uniform reduction in the likelihood of mentioning each content unit in PCA. Instead, certain content units had a much lower chance of being verbalized. Of all content units, “fisherman,” a small, central feature of the WAB Picnic scene, was the least likely to be mentioned by a patient with PCA compared with healthy controls (*r* = −0.85, *p* < 0.001). A few content units had a numerically higher, though not statistically significant, likelihood of being mentioned by patients with PCA compared to healthy individuals, such as “clouds” (*r* = 0.18, *p* = 0.186). Figure 3 is the artistic rending we developed to show the rate at which patients with PCA mention each content unit. We then compared the total number of content units retrieved across the two groups. Overall, PCA patients retrieved fewer content units (mean = 7.20 ± 5.63) compared to healthy individuals (mean = 16.28 ± 4.41) [*t*(45.21) = 6.52, *p* < 0.001]. Lastly, patients with PCA had a lower likelihood of reporting the overall theme of the picture (i.e., mentioning the word “picnic”) (mean = 0.16 ± 0.37), than healthy individuals did (mean = 0.90 ± 0.31) [*t*(46.77), *p* < 0.001].

### Diagnostic classification

**Figure 4:**
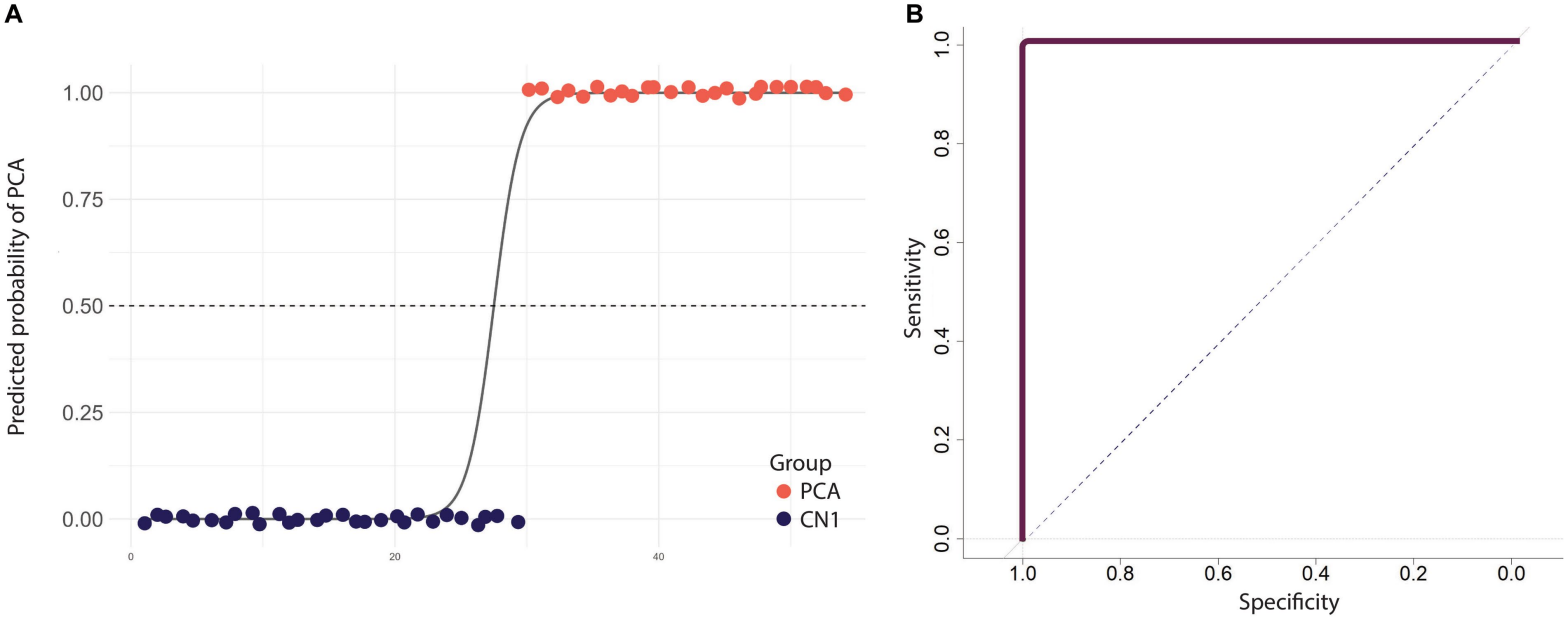
Diagnostic performance of a model that distinguishes PCA patients from healthy individuals using linguistic features from the picture description task. **(A)** The scatter plot shows the predicted probabilities of classifying an individual as having PCA using selected linguistic features. Each point represents an individual participant, with colors indicating the group. The sigmoid curve illustrates the general trend of predicted probabilities. The dashed black line at the predicted probability of 0.5 serves as a decision threshold to classify between PCA patients and healthy individuals. Points are jittered vertically for better visualization. **(B)** ROC curve illustrating the performance of the logistic regression model in discriminating between PCA patients and healthy individuals, with an AUC value of 1 indicating the model’s overall perfect accuracy.

We used binary logistic regression to classify PCA and healthy individuals. Our predictor variables consisted of word frequency, word utterance latency, relational words, the total number of content units, and the probability of mentioning “picnic.” As the sixth variable, we included the probability of mentioning “fisherman” because this content unit had the highest correlation with the group designation, likely due to its visuospatial processing demands. The average accuracy of the model was 98.15% after leave-one-out cross-validation. The average precision across all iterations was found to be 0.96, which means that, on average, 96% of the predicted positive cases were actual positive cases. Moreover, the model demonstrated an average recall of 1, indicating that it successfully identified all the positive cases from the test data in each iteration. We also evaluated the performance of the model using a Receiver Operating Characteristic (ROC) curve. The Area Under the Curve (AUC) was 1, indicating the perfect discrimination ability of the model (Figure 4). Similar prediction outcomes were achieved after word frequency was excluded as a predictor variable, resulting in the most parsimonious model with the highest prediction accuracy. Removing other variables led to a decline in the model’s prediction accuracy.

## Discussion

Using computational linguistic analysis in PCA, our study illuminated a distinction in language performance between visually-dependent and visually-independent contexts. At the theoretical level, we used this method to show that at least some of the language abnormalities increasingly being identified in PCA are byproducts of visuospatial deficits characteristic of this atypical AD syndrome. On the visually-independent job description task, the characteristics of language production we measured here were not impaired in patients with PCA. Translating our observations to clinical practice, we showed that computational linguistic analysis of a simple picture description task robustly classified nearly all PCA patients as distinct from healthy controls, supporting its value in clinical diagnostic evaluation.

Our work is consistent with studies showing rich connections between networks representing information directly received from senses and information conveyed through spoken language (Damasio, 1989; Ralph et al., 2017; Popham et al., 2021). Unimodal sensory information and abstract language information are combined at multiple points across the cortex, such as inferior parietal lobule (comprising the angular and supramarginal gyri) and large swaths of posterolateral temporal cortex (Mesulam, 2000; Devereux et al., 2013), many of which can be affected in PCA. Therefore, language abnormalities in PCA may arise for at least two potential reasons. First, the neurodegeneration of PCA may extend beyond visuospatial areas to encompass regions involved in abstract language processing. In support of this hypothesis, evidence suggests that the brain regions affected in PCA overlap with those critical for word retrieval (Vonk et al., 1991; Migliaccio et al., 2009; Warren et al., 2012; Ossenkoppele et al., 2015; Leyton et al., 2017). Alternatively, language anomalies may arise as a consequence of visuospatial deficits hindering the transfer of necessary sensory information for amodal language processing.

While these two possibilities are not mutually exclusive, our results suggest that language impairments might be largely secondary to visuospatial dysfunction. In our analysis comparing a variety of speech and language properties of the narratives produced when PCA patients describe a complex visual scene versus a recounting of their primary occupation from memory, we observed speech and language impairments in only the visually dependent picture description task. We believe the increased word frequency and word utterance latency in the picture description task reflect visual deficits in object recognition. Similarly, the reduced use of spatial relational words may reflect the patients’ difficulty processing spatial relations between elements of the picnic scene. The absence of abnormalities in word frequency, word utterance latency, and relational words in the job description task provides evidence that these language impairments do not stem from an intrinsic deficit in the language system in PCA.

Since most daily communication is a blend of visuospatial cognition, episodic memory, and other cognitive domains, we anticipate that an analysis of everyday speech would reveal linguistic deficiencies proportionate to the visuospatial load of its content. This expectation aligns with prior research reporting linguistic anomalies in participants recounting their recent holiday, an account that naturally encompasses the visuospatial processing of a recent event, such as where they went and what they saw (Crutch et al., 2013). Relatedly, it has been shown that the autobiographical narratives of patients with PCA have diminished spatiotemporal and perceptual details compared to healthy individuals. This finding likely stems from the impaired access of PCA patients to visual information, which plays a crucial role in constructing autobiographical memories (Ahmed et al., 2018). Furthermore, the narratives of patients with PCA have been shown to be spatially fragmented when they were asked to imagine and describe commonplace scenes (Ramanan et al., 2018). These findings provide converging evidence for the hypothesis that language reveals the visuospatial impairments of PCA proportionate to the visuospatial load of the content.

Another consideration in interpreting these task differences is that the picture description task required the use of specific linguistic elements representing the specific visual stimulus. It is also possible that when given fewer constraints in the job description task, individuals had the freedom to choose a potentially more familiar and more easily accessible language. Our explanation of the underlying language abnormalities in PCA is consistent with findings that showed a striking discrepancy between visual and verbal comprehension tasks in this population (Rogers et al., 2006). Our conclusion is also synergistic with results reporting very mild impairment in semantic memory in PCA, indicating that the apparent semantic impairment in these conditions may be secondary to visual impairment (Rogers et al., 2006).

Based on the observation that language is a sensitive indicator of visuospatial impairments of PCA, we performed an in-depth content unit analysis of language elicited through the picture description task. First, we measured the probability of verbalizing each content unit of the picture. The most distinguishing feature between PCA patients and healthy individuals was the probability of mentioning the “fisherman.” While 93% of healthy participants mentioned this content unit, only 8% of PCA patients did so. This discrepancy could be attributed to the smaller size of this element in the picture. In addition, multiple elements are superimposed in the location of this content unit. Numerically, though not significantly, certain items, such as “clouds,” had a higher likelihood of being mentioned by PCA patients than healthy individuals. This type of analysis provides a naturalistic way of identifying the visuospatial elements that are particularly challenging for PCA patients and could help clinicians devise rehabilitative strategies to alleviate these challenges. Moreover, we observed that PCA patients often missed describing the overarching theme of the image (“picnic”), even when they identified certain components related to it (“basket”). We believe this finding represents the effects of simultagnosia, which prevents many PCA patients from grasping the integrated theme of a composite visual entity.

Finally, when specific quantitative language metrics were employed to differentiate PCA patients from healthy individuals, our predictive model achieved a high level of performance, as evidenced by an AUC of 1 and an accuracy rate of 98.15%. Automating this linguistic evaluation from an easily acquired speech sample would facilitate the integration of measures like this into digital healthcare infrastructure, which a wide array of healthcare providers could potentially use once trained. This work extends our prior research, which used narrative data from a simple picture description task for a high accuracy classification of the three primary progressive aphasia variants (Rezaii et al., 2022, 2023a,b). Future studies are needed to directly analyze the sensitivity and specificity of language predictors across a wide range of patient populations, examine the neurobiological underpinning of linguistic indicators of PCA, and address some of the limitations of this study. These limitations include the absence of neuropsychological scores for participants recruited via the MTurk online platform and the lack of normative data for tests used to determine the severity of PCA symptoms. Ultimately, these advancements will facilitate early diagnosis of PCA as well as monitoring response to disease-modifying, rehabilitative, or other therapies in this underserved atypical variant of AD.

## Data availability statement

The raw data supporting the conclusions of this article will be made available by the authors, without undue reservation.

## Ethics statement

The studies involving humans were approved by Mass General Brigham Human Research Committee Institutional Review Board in Boston, Massachusetts. The studies were conducted in accordance with the local legislation and institutional requirements. The participants provided their written informed consent to participate in this study.

## Author contributions

NR: Conceptualization, Data curation, Formal analysis, Funding acquisition, Investigation, Methodology, Project administration, Resources, Software, Supervision, Validation, Visualization, Writing – original draft, Writing – review & editing. DH: Data curation, Writing – review & editing. MQ: Data curation, Writing – review & editing. BW: Data curation, Writing – review & editing. SM: Data curation, Resources, Writing – review & editing. BD: Conceptualization, Data curation, Funding acquisition, Investigation, Methodology, Project administration, Resources, Supervision, Writing – original draft, Writing – review & editing. DP: Conceptualization, Data curation, Formal analysis, Funding acquisition, Investigation, Methodology, Project administration, Resources, Supervision, Writing – original draft, Writing – review & editing.

## References

[ref1] Ahmed S. Irish M. Loane C. Baker I. Husain M. Thompson S.. (2018). Association between precuneus volume and autobiographical memory impairment in posterior cortical atrophy: beyond the visual syndrome. NeuroImage Clin. 18, 822–834. doi: 10.1016/j.nicl.2018.03.008 29876268 PMC5988022

[ref2] Aliko S. Wang B. Small S. L. Skipper J. I. (2023). The entire brain, more or less is at work: ‘language regions’ are artefacts of averaging. bio Rxiv. doi: 10.1101/2023.09.01.555886v1

[ref3] Alladi S. Xuereb J. Bak T. Nestor P. Knibb J. Patterson K.. (2007). Focal cortical presentations of Alzheimer’s disease. Brain J. Neurol. 130, 2636–2645. doi: 10.1093/brain/awm213 17898010

[ref5] Benson D. F. Davis R. J. Snyder B. D. (1988). Posterior cortical atrophy. Arch. Neurol. 45, 789–793. doi: 10.1001/archneur.1988.00520310107024 3390033

[ref6] Binder J. R. Desai R. H. (2011). The neurobiology of semantic memory. Trends Cogn. Sci. 15, 527–536. doi: 10.1016/j.tics.2011.10.001, PMID: 22001867 PMC3350748

[ref7] Bonferroni C. E. (1935). “Il calcolo delle assicurazioni su gruppi di teste” in Studi in Onore del Professore Salvatore Ortu Carboni. G. Bardi ed Rome, Italy:Tipografia del Senato del dott. 13–60.

[ref8] Cho H. Mundada N. S. Apostolova L. G. Carrillo M. C. Shankar R. Amuiri A. N.. (2023). Amyloid and tau-PET in early-onset AD: baseline data from the longitudinal early-onset Alzheimer’s disease study (LEADS). Alzheimers Dement.:13453. doi: 10.1002/alz.13453 PMC10807231 37690109

[ref9] Cloud Speech-to-Text API v1 - Package cloud. Go client library|Google Cloud [Internet]. Available at: www.google.com/go/speech/apiv1(v1.19.0) and https://cloud.google.com/go/docs/reference/cloud.google.com/go/speech/latest/apiv1

[ref10] Cordella C. Quimby M. Touroutoglou A. Brickhouse M. Dickerson B. C. Green J. R. (2019). Quantification of motor speech impairment and its anatomic basis in primary progressive aphasia. Neurology 92, e1992–e2004. doi: 10.1212/WNL.0000000000007367, PMID: 30944238 PMC6511075

[ref11] Crutch S. J. Lehmann M. Warren J. D. Rohrer J. D. (2013). The language profile of posterior cortical atrophy. J. Neurol. Neurosurg. Psychiatry 84, 460–466. doi: 10.1136/jnnp-2012-303309, PMID: 23138762 PMC4667396

[ref12] Crutch S. J. Schott J. M. Rabinovici G. D. Murray M. Snowden J. S. van der Flier W. M.. (2017). Consensus classification of posterior cortical atrophy. Alzheimers Dement. J. Alzheimers Assoc. 13, 870–884. doi: 10.1016/j.jalz.2017.01.014, PMID: 28259709 PMC5788455

[ref13] Cui Y. Liu Y. Yang C. Cui C. Jing D. Zhang X.. (2022). Brain structural and functional anomalies associated with simultanagnosia in patients with posterior cortical atrophy. Brain Imaging Behav. 16, 1148–1162. doi: 10.1007/s11682-021-00568-8, PMID: 34787788 PMC9107404

[ref14] Damasio A. R. (1989). Time-locked multiregional retroactivation: a systems-level proposal for the neural substrates of recall and recognition. Cognition 33, 25–62. doi: 10.1016/0010-0277(89)90005-X, PMID: 2691184

[ref15] Delis DC Kramer JH Kaplan E Thompkins BAO. California verbal learning test-second edition (CVLT-II). San Antonio, TX: Psychological Corporation; (2000) 91

[ref16] Devereux B. J. Clarke A. Marouchos A. Tyler L. K. (2013). Representational similarity analysis reveals commonalities and differences in the semantic processing of words and objects. J. Neurosci. 33, 18906–18916. doi: 10.1523/JNEUROSCI.3809-13.2013, PMID: 24285896 PMC3852350

[ref17] Dickerson B. C. McGinnis S. M. Xia C. Price B. H. Atri A. Murray M. E.. (2017). Approach to atypical Alzheimer’s disease and case studies of the major subtypes. CNS Spectr. 22, 439–449. doi: 10.1017/S109285291600047X, PMID: 28196556 PMC5557706

[ref18] Fleisher A. S. Pontecorvo M. J. Devous M. D. Sr. Lu M. Arora A. K. Truocchio S. P.. (2020). Positron emission tomography imaging with [18F] flortaucipir and postmortem assessment of Alzheimer disease Neuropathologic changes. JAMA Neurol. 77, 829–839. doi: 10.1001/jamaneurol.2020.0528, PMID: 32338734 PMC7186920

[ref19] Godfrey JJ Holliman EC McDaniel J. SWITCHBOARD: telephone speech corpus for research and development. In Proceedings of the 1992 IEEE international conference on acoustics, speech and signal processing San Francisco, CA, USA: IEEE Computer Society (1992). 517–520

[ref20] Herrera-Guzmán I. Peña-Casanova J. Lara J. P. Gudayol-Ferré E. Böhm P. (2004). Influence of age, sex, and education on the visual object and space perception battery (VOSP) in a healthy normal elderly population. Clin. Neuropsychol. 18, 385–394. doi: 10.1080/1385404049052421, PMID: 15739810

[ref21] Humphreys GW Riddoch JM. (1993). BORB: Birmingham object recognition battery. Available at https://www.routledge.com/BORB-Birmingham-Object-Recognition-Battery/Riddoch-Humphreys/p/book/9780863773150

[ref22] Huth A. G. de Heer W. A. Griffiths T. L. Theunissen F. E. Gallant J. L. (2016). Natural speech reveals the semantic maps that tile human cerebral cortex. Nature 532, 453–458. doi: 10.1038/nature17637, PMID: 27121839 PMC4852309

[ref23] Josephy-Hernandez S. Rezaii N. Jones A. Loyer E. Hochberg D. Quimby M.. (2023). Automated analysis of written language in the three variants of primary progressive aphasia. Brain Commun. 5:fcad 202. doi: 10.1093/braincomms/fcad202, PMID: 37539353 PMC10396070

[ref24] Kertesz A Kertesz A Raven JC, Psych Corp (firm). WAB-R: Western aphasia battery-revised. San Antonio, TX: Psych Corp (2007)

[ref25] Lehmann M. Crutch S. J. Ridgway G. R. Ridha B. H. Barnes J. Warrington E. K.. (2011). Cortical thickness and voxel-based morphometry in posterior cortical atrophy and typical Alzheimer’s disease. Neurobiol. Aging 32, 1466–1476. doi: 10.1016/j.neurobiolaging.2009.08.017, PMID: 19781814

[ref26] Levine D. N. Lee J. M. Fisher C. M. (1993). The visual variant of Alzheimer’s disease: a clinicopathologic case study. Neurology 43, 305–313. doi: 10.1212/WNL.43.2.305 8437694

[ref27] Leyton C. E. Hodges J. R. Piguet O. Ballard K. J. (2017). Common and divergent neural correlates of anomia in amnestic and logopenic presentations of Alzheimer’s disease. Cortex J Devoted Study Nerv. Syst. Behav. 86, 45–54. doi: 10.1016/j.cortex.2016.10.019, PMID: 27875715

[ref4] McFee B Raffel C. Liang D. Ellis D. P. W. McVicar M. Battenbergk E. (2015) “librosa: Audio and music signal analysis in python.” In Proceedings of the 14th python in science conference. 18–25.

[ref28] McMonagle P. Deering F. Berliner Y. Kertesz A. (2006). The cognitive profile of posterior cortical atrophy. Neurology 66, 331–338. doi: 10.1212/01.wnl.0000196477.78548.db 16476930

[ref29] Mesulam M. M. (2000). (ed.) “Behavioral neuroanatomy” in Principles of behavioral and cognitive neurology. 2nd ed (Oxford, New York: Oxford University Press)

[ref30] Migliaccio R. Agosta F. Rascovsky K. Karydas A. Bonasera S. Rabinovici G. D.. (2009). Clinical syndromes associated with posterior atrophy: early age at onset AD spectrum. Neurology 73, 1571–1578. doi: 10.1212/WNL.0b013e3181c0d427, PMID: 19901249 PMC2777069

[ref31] Nicholas L. E. Brookshire R. H. (1993). A system for quantifying the informativeness and efficiency of the connected speech of adults with aphasia. J. Speech Hear. Res. 36, 338–350. doi: 10.1044/jshr.3602.338, PMID: 8487525

[ref32] Ossenkoppele R. Cohn-Sheehy B. I. La Joie R. Vogel J. W. Möller C. Lehmann M.. (2015). Atrophy patterns in early clinical stages across distinct phenotypes of Alzheimer’s disease. Hum. Brain Mapp. 36, 4421–4437. doi: 10.1002/hbm.22927, PMID: 26260856 PMC4692964

[ref33] Popham S. F. Huth A. G. Bilenko N. Y. Deniz F. Gao J. S. Nunez-Elizalde A. O.. (2021). Visual and linguistic semantic representations are aligned at the border of human visual cortex. Nat. Neurosci. 24, 1628–1636. doi: 10.1038/s41593-021-00921-6 34711960

[ref34] Putcha D. Brickhouse M. Touroutoglou A. Collins J. A. Quimby M. Wong B.. (2019). Visual cognition in non-amnestic Alzheimer’s disease: relations to tau, amyloid, and cortical atrophy. Neuro Image Clin. 23:101889. doi: 10.1016/j.nicl.2019.101889 PMC6562373 31200149

[ref35] Putcha D. Dickerson B. C. Brickhouse M. Johnson K. A. Sperling R. A. Papp K. V. (2020). Word retrieval across the biomarker-confirmed Alzheimer’s disease syndromic spectrum. Neuropsychologia 140:107391. doi: 10.1016/j.neuropsychologia.2020.107391, PMID: 32057937 PMC7085933

[ref36] Putcha D. McGinnis S. M. Brickhouse M. Wong B. Sherman J. C. Dickerson B. C. (2018). Executive dysfunction contributes to verbal encoding and retrieval deficits in posterior cortical atrophy. Cortex J. Devoted Study Nerv. Syst. Behav. 106, 36–46. doi: 10.1016/j.cortex.2018.04.010, PMID: 29864594 PMC6120771

[ref37] Qi P Zhang Y Zhang Y Bolton J Manning CD. Stanza: a Python natural language processing toolkit for many human languages. In Proceedings of the 58th annual meeting of the Association for Computational Linguistics: System demonstrations. (2020)

[ref38] Rabinovici G. D. Furst A. J. Alkalay A. Racine C. A. O’Neil J. P. Janabi M.. (2010). Increased metabolic vulnerability in early-onset Alzheimer’s disease is not related to amyloid burden. Brain 133, 512–528. doi: 10.1093/brain/awp326, PMID: 20080878 PMC2858015

[ref39] Rabinovici G. D. Rosen H. J. Alkalay A. Kornak J. Furst A. J. Agarwal N.. (2011). Amyloid vs FDG-PET in the differential diagnosis of AD and FTLD. Neurology 77, 2034–2042. doi: 10.1212/WNL.0b013e31823b9c5e, PMID: 22131541 PMC3236517

[ref40] Ralph M. A. L. Jefferies E. Patterson K. Rogers T. T. (2017). The neural and computational bases of semantic cognition. Nat. Rev. Neurosci. 18, 42–55. doi: 10.1038/nrn.2016.150, PMID: 27881854

[ref41] Ramanan S. Alaeddin S. Goldberg Z. Strikwerda-Brown C. Hodges J. R. Irish M. (2018). Exploring the contribution of visual imagery to scene construction – evidence from posterior cortical atrophy. Cortex 106, 261–274. doi: 10.1016/j.cortex.2018.06.016, PMID: 30059847

[ref42] Renner J. A. Burns J. M. Hou C. E. McKeel D. W. Storandt M. Morris J. C. (2004). Progressive posterior cortical dysfunction: a clinicopathologic series. Neurology 63, 1175–1180. doi: 10.1212/01.WNL.0000140290.80962.BF 15477534

[ref43] Rezaii N. Mahowald K. Ryskin R. Dickerson B. Gibson E. (2022). A syntax-lexicon trade-off in language production. Proc. Natl. Acad. Sci. U. S. A 119:e2120203119. doi: 10.1073/pnas.2120203119, PMID: 35709321 PMC9231468

[ref44] Rezaii N. Michaelov J. Josephy-Hernandez S. Ren B. Hochberg D. Quimby M.. (2023a). Measuring sentence information via Surprisal: theoretical and clinical implications in nonfluent aphasia. Ann. Neurol. 94, 647–657. doi: 10.1002/ana.26744, PMID: 37463059 PMC10543558

[ref45] Rezaii N. Quimby M. Wong B. Hochberg D. Brickhouse M. Touroutoglou A.. (2023b). Using generative artificial intelligence to classify primary progressive aphasia from connected speech. Med Rxiv. doi: 10.1101/2023.12.22.23300470v1 PMC11370793 38912855

[ref46] Rezaii N. Ren B. Quimby M. Hochberg D. Dickerson B. C. (2023c). Less is more in language production: an information-theoretic analysis of agrammatism in primary progressive aphasia. Brain Commun. 5:fcad136. doi: 10.1093/braincomms/fcad136, PMID: 37324242 PMC10263269

[ref47] Rezaii N. Wolff P. Price B. H. (2022). Natural language processing in psychiatry: the promises and perils of a transformative approach. Br. J. Psychiatry 220, 251–253. doi: 10.1192/bjp.2021.188, PMID: 35048814

[ref48] Rogers T. T. Ivanoiu A. Patterson K. Hodges J. R. (2006). Semantic memory in Alzheimer’s disease and the frontotemporal dementias: a longitudinal study of 236 patients. Neuropsychology 20, 319–335. doi: 10.1037/0894-4105.20.3.319 16719625

[ref49] Shirk S. D. Mitchell M. B. Shaughnessy L. W. Sherman J. C. Locascio J. J. Weintraub S.. (2011). A web-based normative calculator for the uniform data set (UDS) neuropsychological test battery. Alzheimers Res. Ther. 3:32. doi: 10.1186/alzrt94, PMID: 22078663 PMC3308021

[ref50] Singh T. D. Josephs K. A. Machulda M. M. Drubach D. A. Apostolova L. G. Lowe V. J.. (2015). Clinical, FDG and amyloid PET imaging in posterior cortical atrophy. J. Neurol. 262, 1483–1492. doi: 10.1007/s00415-015-7732-5, PMID: 25862483 PMC4469094

[ref51] Sonni I. Lesman Segev O. H. Baker S. L. Iaccarino L. Korman D. Rabinovici G. D.. (2020). For the Alzheimer's disease neuroimaging initiative evaluation of a visual interpretation method for tau-PET with 18F-flortaucipir. DADM 12:e12133. doi: 10.1002/dad2.12133, PMID: 33313377 PMC7699207

[ref52] Stern RA White T. Neuropsychological assessment battery. Psychological assessment resources. Lutz, FL; (2003). Available at: https://www.parinc.com/Products?pkey=260

[ref53] Strauss E. Sherman E. M. S. Spreen O. (2006). A compendium of neuropsychological tests: Administration, norms, and commentary New York, USA: Oxford University Press, 1235.

[ref54] Tang-Wai D. F. Graff-Radford N. R. Boeve B. F. Dickson D. W. Parisi J. E. Crook R.. (2004). Clinical, genetic, and neuropathologic characteristics of posterior cortical atrophy. Neurology 63, 1168–1174. doi: 10.1212/01.WNL.0000140289.18472.15, PMID: 15477533

[ref55] Tomaszewski Farias S. Mungas D. Harvey D. J. Simmons A. Reed B. R. Decarli C. (2011). The measurement of everyday cognition: development and validation of a short form of the everyday cognition scales. Alzheimers Dement. J. Alzheimers Assoc. 7, 593–601. doi: 10.1016/j.jalz.2011.02.007, PMID: 22055976 PMC3211103

[ref56] Villeneuve S. Rabinovici G. D. Cohn-Sheehy B. I. Madison C. Ayakta N. Ghosh P. M.. (2015). Existing Pittsburgh compound-B positron emission tomography thresholds are too high: statistical and pathological evaluation. Brain 138, 2020–2033. doi: 10.1093/brain/awv112, PMID: 25953778 PMC4806716

[ref57] Vonk J. M. J. Rizvi B. Lao P. J. Budge M. Manly J. J. Mayeux R.. (1991). Letter and category fluency performance correlates with distinct patterns of cortical thickness in older adults. Cereb Cortex N Y N 29, 2694–2700. doi: 10.1093/cercor/bhy138, PMID: 29893804 PMC6519688

[ref58] Warren J. D. Fletcher P. D. Golden H. L. (2012). The paradox of syndromic diversity in Alzheimer disease. Nat. Rev. Neurol. 8, 451–464. doi: 10.1038/nrneurol.2012.135, PMID: 22801974

[ref59] Weintraub S. Besser L. Dodge H. H. Teylan M. Ferris S. Goldstein F. C.. (2018). Version 3 of the Alzheimer disease centers’ neuropsychological test battery in the uniform data set (UDS). Alzheimer Dis. Assoc. Disord. 32, 10–17. doi: 10.1097/WAD.0000000000000223, PMID: 29240561 PMC5821520

[ref60] Whitwell J. L. Jack C. R. Kantarci K. Weigand S. D. Boeve B. F. Knopman D. S.. (2007). Imaging correlates of posterior cortical atrophy. Neurobiol. Aging 28, 1051–1061. doi: 10.1016/j.neurobiolaging.2006.05.026, PMID: 16797786 PMC2734142

[ref61] Wong B. Lucente D. E. Mac Lean J. Padmanabhan J. Quimby M. Brandt K. D.. (2019). Diagnostic evaluation and monitoring of patients with posterior cortical atrophy. Neurodegener. Dis. Manag. 9, 217–239. 31392920 10.2217/nmt-2018-0052 PMC6949516

