# Supplementary material for: Language uncovers visuospatial dysfunction in posterior cortical atrophy: a natural language processing approach
Source: Front Neurosci. 2024 Feb 6;18:1342909. doi: 10.3389/fnins.2024.1342909 (PMC10876777; doi:10.3389/fnins.2024.1342909)
Supplement: Supplementary file 1 [file Data_Sheet_1.PDF]

Supplementary Materials.

To ensure that the language findings of this study were not confounded by the difference in participants' years of education (YOE), we conducted two types of analyses.

1. Correlational analyses.

We found no statistically significant correlations between the language variables of interest and YOE across the two tasks.

In the picnic description task, there was no significant correlation between the participants' YOE and word frequency ($r = 0.18$, $p = 0.36$), word onset latency ($r = -0.11$, $p = 0.56$), and relational words ($r = -0.04$, $p = 0.82$).

Similarly, in the job description task, there was no significant correlation between the participants' YOE and word frequency ($r = 0.13$, $p = 0.58$), word onset latency ($r = -0.08$, $p = 0.74$), and relational words ($r = 0.07$, $p = 0.75$).

2. Subgroup analyses that matched the participants' YOE.

Here, we repeat the analyses of the main manuscript in subgroups of participants with matched YOE.

Picture description task. Similar to the main results, patients with PCA used higher frequency words (6.51) than healthy controls (5.87), $t(44.16) = -4.45$, $p < 0.001$. Patients with PCA had higher word utterance latency (0.40) than healthy controls (0.03), $t(23.29) = -3.20$, $p = 0.004$. Patients with PCA used fewer relational words (0.08) than healthy controls (0.09), $t(44.26) = 2.09$, $p = 0.04$.

Job description task. Similar to the main results, there was no significant difference between PCA(6.25) and healthy individuals (6.26) in terms of word frequency ($t(34.30) = 0.07$, $p = 0.95$). There was no significant difference between PCA(0.10) and healthy individuals (0.05) in terms of word onset latency ($t(19.65) = -0.877$, $p = 0.39$). We also found no significant difference between PCA(0.10) and healthy individuals (0.09) with respect to relational words ($t(28.77) = -1.177$, $p = 0.25$).
